# Extended and Fully Automated Newborn Screening Method for Mass Spectrometry Detection

**DOI:** 10.3390/ijns4010002

**Published:** 2017-12-29

**Authors:** Stefan Gaugler, Jana Rykl, Irene Wegner, Tamara von Däniken, Ralph Fingerhut, Götz Schlotterbeck

**Affiliations:** 1 CAMAG, Sonnenmattstr. 11, 4132 Muttenz, Switzerland; 2 Shimadzu Schweiz GmbH, Römerstrasse 3, 4153 Reinach, Switzerland; 3 Institute of Chemistry and Bioanalytics, University of Life Sciences, University of Applied Sciences Northwestern Switzerland FHNW, Gründenstr. 40, 4132 Muttenz, Switzerland; 4 Swiss Newborn Screening Laboratory, Division of Metabolism and Children’s Research Center, University Children’s Hospital Zurich, Steinwiesstrasse 75, 8032 Zurich, Switzerland

**Keywords:** newborn screening (NBS), dried blood spots (DBS), automation

## Abstract

A new and fully automated newborn screening method for mass spectrometry was introduced in this paper. Pathological relevant amino acids, acylcarnitines, and certain steroids are detected within 4 min per sample. Each sample is treated in an automated and standardized workflow, where a mixture of deuterated internal standards is sprayed onto the sample before extraction. All compounds showed good linearity, and intra- and inter-day variation lies within the acceptance criteria (except for aspartic acid). The described workflow decreases analysis cost and labor while improving the sample traceability towards good laboratory practice.

## 1. Introduction

Newborn screening (NBS) is a public health program provided by most of the countries around the world aimed at screening newborns for a list of serious genetic and metabolic disorders [1,2,3,4,5,6]. Early diagnosis of these conditions can help prevent their further development, which untreated often results in brain damage, organ damage, and even death. A routine neonatal screening procedure requires that a health professional takes a few drops of blood from the baby’s heel, applies them onto special filter paper and sends such prepared samples to a laboratory for a number of analytical tests [7]. The sample preparation before analysis may be labor-intensive and time-consuming when processed with traditional “punch-and-elute” methodology. To date probably all NBS programs use punchers (manual, semi-automated, or totally automated) which transfer a 3.2 mm punch of the dried blood spot (DBS) into a microtiter plate. Although most puncher software programs register which sample has been punched into which well of the respective microtiter plate, there is no control as to whether a DBS has flipped out of the respective well due to static electricity.

Application of automated DBS card handling systems, which are connected to mass spectrometry analyzers, offers a modern and fast approach where a circular area of the DBS is directly eluted from the filter paper card without any punching. A panel of relevant biomarkers for the mass spectrometry newborn screening, including amino acids and acylcarnitines, was chosen for this study. Additionally, a steroid panel has been added to integrate new biomarkers which may lead to clinical relevant data. Automation for this study was implemented by the CAMAG DBS-MS 500 equipment.

Today, with the advancement of suitable instrumentation, more and more analytes are transferred from being detected by biological assays into the mass spectrometry screening panel with the advancement of those instruments [6,8,9,10,11].

## 2. Materials and Methods

### 2.1. Chemicals

Gradient grade water and LC-MS grade methanol for liquid chromatography plus the non LC-MS grade rinsing solvents 2-propanol and acetonitrile as well as formic acid (puriss.) and ammonia fluoride (puriss.) were purchased from Carl Roth (Carl Roth, Rothenfels, Germany). A collection of all l-amino acids and carnitine, acetylcarnitine, propionylcarnitine, butyrylcarnitine, valerylcarnitine, hexanoylcarnitine, octanoylcarnitine, decanoylcarnitine, lauroylcarnitine, myristoylcarnitine, palmitoylcarnitine and stearoylcarnitine, were purchased from Sigma-Aldrich (St. Louis, MO, USA).

The deuterated internal standards (IS) Cortisol ^2^H_4_, Progesterone ^2^H_9_, 17-Hydroxyprogesterone ^2^H_8_, 11-Deoxycortisol ^2^H_5_, 21-Deoxycortisol ^2^H_8_, Androstenedione ^13^C_3_, Corticosterone ^2^H_4_, 11-Deoxycorticosterone ^13^C_3_ were also products of Sigma-Aldrich (St. Louis, MO, USA). The deuterated amino acids and acylcarnitines were obtained from the MassChrom^®^ Amino Acids and Acylcarnitines from Dried Blood kit from Chromsystems (Munich, Germany). Dried blood spot cards (TFN filter paper) were provided by CAMAG (Muttenz, Switzerland). Fresh whole blood was obtained from the local blood donation center (Basel, Switzerland).

### 2.2. Analytical Methods

#### Analytical Materials and Methods

Chromatography was performed on a modular HPLC system from Shimadzu (Kyoto, Japan); it contained a system controller (CBM-20A), two Nexera X2 pumps, a degasser (DGU-20ASR), and a column oven (CTO-20AC). Automated extractions were carried out with a DBS-MS 500 (CAMAG, Muttenz, Switzerland). Analytes were separated on a Shim-pack GIST (4.6 × 50 mm, 5 μm STEAROYL, 227-30017-3) analytical column (Shimadzu, Kyoto, Japan). A filter frit (KrudKatcher Ultra, Phenomenex, Torrance, CA, USA) was connected upstream to the analytical column. Mobile phase A consisted of water plus 0.1% formic acid and 2 mM ammonia fluoride, while methanol supplemented with 0.1% formic acid and 2 mM ammonia fluoride was used as mobile phase B. The following stepwise gradient was applied: 40% A (0–1.0 min), 40–90% A (1.0–2.0 min), 90% A (2.0–3.0 min), and 40% A (3.01–4.0 min). The flow rate was set at 1.0 mL/min at 40 °C. The HPLC liquid stream was connected to an 8060 tandem mass spectrometer (Shimadzu, Kyoto, Japan). The mass transitions and compound specific settings were included in Table S1.

### 2.3. DBS-MS 500 Settings

The extraction solvent on the DBS-MS 500 (CAMAG, Switzerland) was a mixture of methanol and water (70:30 *v/v*) and was connected to extraction port E1/R3. The wash solution, consisting of methanol, acetonitrile, 2-propanol and water (25:25:25:25, *v/v/v/v*), with 0.1% formic acid, was connected to the rinsing bottle R1. The internal standard mix was connected to IS2, and IS4. IS wash was filled with methanol. The system was prepared by priming methanol through the internal standard port (10 cycles) followed by 2 cycles of IS2. The extraction head was cleaned in an ultra sound bath at 40 °C for 10 min prior to a large set of analyses. The extraction solvent was primed for 5 cycles and the rinsing solvents were flushed for 1 min (this process is an automated system prime method). The DBS cards were photographed with the built-in camera of the DBS-MS 500 before and after each extraction to check for the presence of a blood spot and to adjust the extraction head to the center of each spot. The Chronos for CAMAG software automatically recognized inadequate dried blood spots based on their roundness, diameter, and area. Inadequate DBS were excluded from analysis. Twenty microliters of internal standard were sprayed in a homogenous layer onto each spot. After a 20 s drying time, the samples were extracted with a volume of 20 μL and a 200 μL/min flow rate. To complete the automated DBS extraction cycle, the system was rinsed for 20 s with R1 [12].

### 2.4. DBS-MS 500 and LC-MS/MS Interface

**Figure 1:**
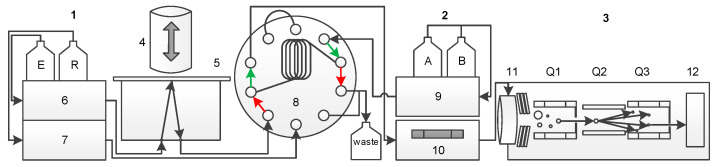
Flow scheme of the automated DBS-LC-MS/MS approach: 1, Automated dried blood spot (DBS) system; 2, HPLC; 3, MS/MS; 4, Extraction head; 5, DBS card; 6, Extraction pump; 7, Rinsing pump; 8, 10-port valve interface; 9, HPLC pump; 10, HPLC oven and analytical column; 11, ESI source; and 12, detector.

The flow scheme of the fully automated card extraction system and the coupled LC-MS/MS is shown in Figure 1. The DBS cards are moved to the extraction unit, where a plunger seals a circular region of the card. The extraction solvent is pumped through this sealed region of the card and loaded into a loop (Figure 1: red arrows). By switching the 10-port valve, the loop volume is connected to the LC-MS/MS flow path (Figure 1: green arrows) and guided to the column and after separation to the tandem MS. Meanwhile, the extraction head is cleaned by a rinsing cycle to avoid carry over.

### 2.5. Sample and Standard Preparation

According to reference values, stock solutions were prepared and spiked to fresh blood in three different amounts to generate four levels in total, including the endogenous level from the male donor. The amino acids, carnitine, and acetylcarnitine were dissolved in water to prepare spike solution A (Table S2). The remaining carnitines were dissolved in methanol (spike B). The steroids were diluted with methanol to 100 μg/mL and mixed (spike C). Spike solutions A, B and C were stored at 4 °C.

#### 2.5.1. Working Standards for LC-MS/MS Tuning

The spike solutions were used for LC-MS/MS method development and tuning of the MRM transitions. Spike A was diluted 100-fold and spike B and C 10-fold prior to injection.

#### 2.5.2. Internal Standards

The deuterated steroids were dissolved in methanol to prepare 100 μg/mL standard solutions. Then, a mix was prepared with a final concentration of 10 μg/mL for all deuterated steroids. A MassChrom^®^ internal standard mix (intended for 50 mL extraction buffer) was dissolved in 4.95 mL methanol and 50 μL of the deuterated steroid mix was added. This solution was used as an internal standard mix on the DBS-MS 500.

#### 2.5.3. Dried Blood Spot Samples

The method should be applicable for using a calibration curve and for the detection via a reference signal, which improves the accuracy of the method. Therefore, 4 calibration levels were prepared. The calibration curve can be drawn using the 4 points and the zero concentration can be derived through the standard addition method. Alternatively, level 2 and 4 can be used as low and high controls when comparing the analyte peak and IS ratio. Freshly collected human blood was obtained from the local blood donation center (Basel, Switzerland). EDTA was used as an anticoagulation agent (vacutainer tubes, BD, Allschwil, Switzerland). Spiked blood samples (prepared according to (Table S3) were gently mixed, after which 50 μL aliquots were spotted by an Eppendorf pipette onto CAMAG DBS cards (CAMAG, Muttenz, Switzerland). DBS cards were dried at room temperature for at least 3 h and were subsequently stored at 4 °C in sealed plastic bags containing desiccants. All DBS were prepared from the same blood source, prepared with the same procedure to neutralize potential hematocrit effect [12,13], which were not investigated in this study.

### 2.6. Quantification Method

All compounds were spiked in three different concentrations, resulting in levels 2, 3, and 4; level 1 reflects the endogenous concentration of the donor blood. Quantification can be performed by the standard addition method using the three calibration points or by comparing the analyte to internal standard peak ratio using level 2 as low and level 4 as high control. Those calibration cards with level 1–4 can directly be used as quality control standards in the developed method. Quantification by comparing the peak ratio is often used in newborn screening labs, whereas the three point calibration is a more widely used method in instrumental analytics [14,15], is accurate [16] and which is applicable on any commercial mass spectrometry software.

### 2.7. Documentation

The documentation process is given by the DBS-MS 500 instrument which takes a picture of the DBS card before and after each extraction to assure sample traceability. All pressures on the DBS card sampler as well as on the LC-MS/MS unit are monitored and documented.

The camera documentation system checks for preset values, where quality control parameters can be integrated. If the quality criteria are not met, the system automatically checks the next spot and continues the preset program. In addition, the camera detects already extracted spots and blocks those to prevent reanalysis. The results and preset criteria from the DBS card shown in Figure S1 are listed in Table S4. The system checks the xy shift of the circle on the card and the blood spot to center the internal standard spray and the extraction spot to the middle of each DBS (to avoid inhomogeneous distribution effects). Further, the inbuilt pressure sensors are monitoring the extraction and rinsing pressure (in this study, the extraction pressure was 0.9 bar and the rinsing pressure 41.5 bar). The maximum extraction pressure is 1.5 bar, depending on the age of the blood sample, and the system can be rinsed with pressure up to 100 bar to prevent carry over.

## 3. Results

### 3.1. LC-MS/MS Method

**Figure 2:**
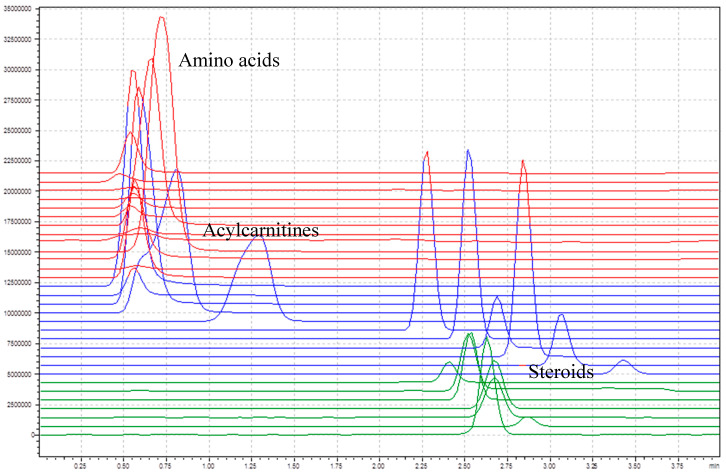
Total ion chromatogram (TIC) of all unlabeled target compounds (amino acids in red, acylcarnitines in blue and steroids in green).

A C18 column was used to have a quick separation of the target compounds. It is important to separate the acylcarnitines and some of the steroid, since they fragment into the same daughter ions. Figure 2 shows the total ion chromatogram of all target compounds without internal standards. The amino acids and the short-chain acylcarnitines (C0–C4) elute in the first section before 0.75 min, the longer chained acylcarnitines (C5–C18) between 0.75 and 4 min, and the steroids between 2.25 and 3 min. The main goal of the column integration was to separate acylcarnitines from steroids, since some of them have similar multiple reaction monitoring (MRM) transitions. Therefore, the chromatographic separation was not further optimized.

### 3.2. Linearity and Storage Conditions

**Figure 3:**
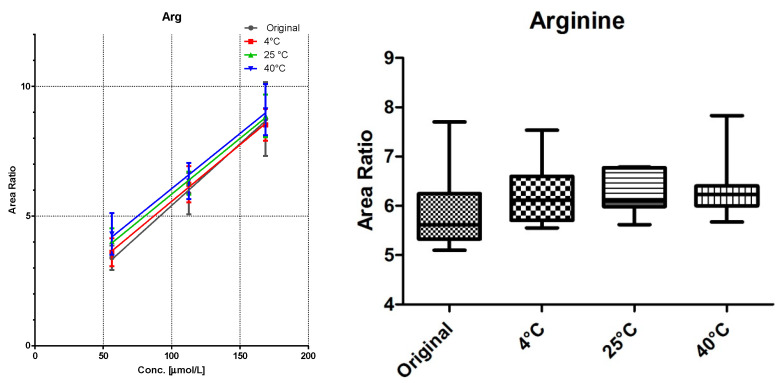
LC-MS/MS analysis of arginine from DBS cards stored at different storage temperatures: (**left**) linearity (mean ± sd of *n* = 7); and (**right**) box and whiskers graph of level 2 with 5–95 percentiles of the same data.

**Figure 4:**
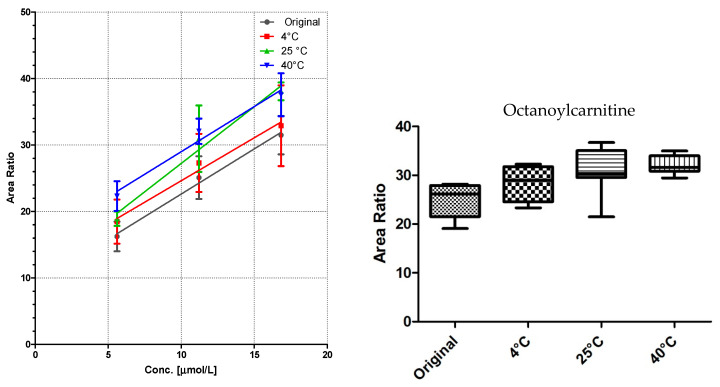
LC-MS/MS analysis of octanoylcarnitine from DBS cards stored at different storage temperatures: (**left**) linearity (mean ± sd of *n* = 7); and (**right**) box and whiskers graph of level 2 with 5–95 percentiles of the same data.

**Figure 5:**
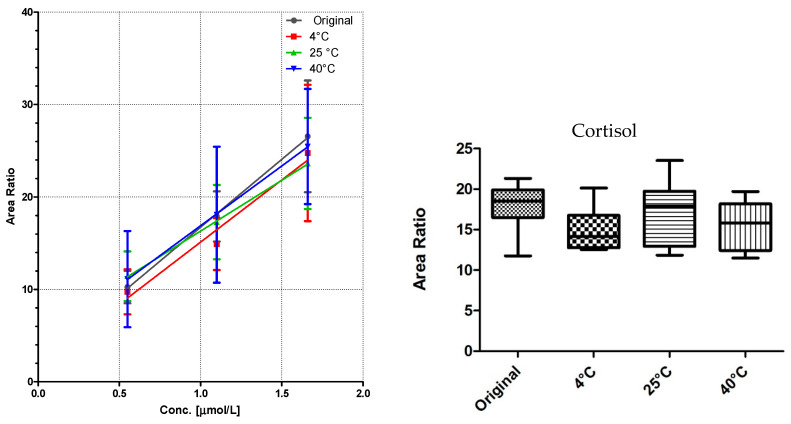
LC-MS/MS analysis of cortisol from DBS cards stored at different storage temperatures: (**left**) linearity (mean ± sd of *n* = 7); and (**right**) box and whiskers graph of level 2 with 5–95 percentiles of the same data.

All DBS samples were measured 10-fold to determine the method robustness and intra-day variations. The samples were then stored for seven days at three different storage conditions: 4 °C in the fridge, at room temperature, and at 40 °C in an oven. Of each substance class, a compound was chosen for visualization (Figure 3, Figure 4 and Figure 5).

Comparison of the data shows no significant differences (calculated by GraphPad Prism software) between the first time point (“original”) and the results from differently stored cards. As seen with octanoylcarnitine (Figure 4), the mean signal intensity may even be higher if stored at elevated temperatures. However, this effect is not significant from the gathered data and has to be monitored more closely in a follow up study.

A sufficient linearity was reached for all compounds (Table 1), with the exception of myristoylcarnitine. The storage temperature within one week has no influence on the calibration function, as shown in Figure 3, Figure 4 and Figure 5.

### 3.3. Intra-Day and Inter-Day Variations

The intra- and inter-day variations as well as the coefficients of determination (R^2^) of the three calibration standards are summarized in Table 1. The experiments performed with the DBS-LC-MS/MS system showed excellent linearity. Performances of accuracy and precision were acceptable according to the objectives of these explorations with all CV and biases <20% with two exceptions for the inter-day variation: aspartic acid and glycine. This is due to a shift in peak height between the two compared time points. In a routine setup, this could be avoided by measuring a quality control card as reference each day of use. In addition, carry over was monitored by injecting blank samples after measuring the high QC level number 4, where the blank signal was less than 1% of the high signal.

## 4. Discussion

The described method follows the trend of the industry towards fully automated processes and also presents a new expanded LC-MS/MS method for newborn screening. In addition to the complete panel of all clinically relevant amino acids and acylcarnitines, a steroid panel was integrated for the first time in a single, fast and fully automated LC-MS/MS method.

The three point calibration curve allows accurate quantification with independent analysis software. The different storage conditions examined in this work had no significant influence on the results and the inter- and intra-day variations are in an acceptable range for a screening method.

The method takes 4 min per sample using the Shim-pack GIST column. Due to the chromatographic separation, the extract is purified, which increases the system robustness. There is no maximum sample amount per column given by the manufacturer, however it is approximated to be plus 1000 samples per column. The total system backpressure was less than 100 bar and the method runs each sample subsequently. Coversely, the traditional punch-and-elute is a batch approach via the 96 well plate format. In contrast to the conventional method, the DBS-MS 500 extracts a centered 4 mm circular area. This does not allow extracting the same DBS twice, however it neutralizes inhomogeneous distribution effects [17] through the software centration of the punching location. In most DBS protocols [3], at least 100 μL extraction solution with internal standard plus sample preparation consumables such as vials and pipette tips are used. The DBS-MS 500 system only uses 20 μL extraction solution and the internal standard is added prior the extraction. To monitor the extraction efficiency and to follow method development guidelines, the internal standard should be added as early as possible into the workflow [18].

The DBS-LC-MS/MS system is capable of handling up to 1200 bar in the analytical circuit and preliminary studies have shown that the process can easily be accelerated to 2.5 min per samples by using denser columns and higher pressure on the system. Bypassing the column is not an option, since the steroids and some acylcarnitines have to be separated for a reliable detection through MRM, since they fragment into the same product ions.

This preliminary study shows the method feasibility and potential of using such automated equipment in the sector of new born screening. To implement such a method, a follow-up study with an extensive validation is planned. In addition, there should be a direct comparison between the punch-and-elute methodology and the direct elution approach described in this study.

Further, the preparation of the calibrators and the quality control cards should be investigated more closely. Basically, the blood can be spiked directly (as shown here), the red blood cells (RBC) can be separated from the plasma, whereas the plasma is then spiked or exchanged with a spiked saline or bovine serum albumin solution, prior remixing with the RBC. The direct addition of spiked solvents may cause hemolysis or protein denaturation above a certain percentage.

## 5. Conclusions

In addition to the complete coverage of all amino acids and acylcarnitines of interest, an additional steroid panel was integrated to allow screening for congenital adrenal hyperplasia currently determined by separate biological assays. This screening method decreases the analysis cost through full automation and an economical use of solvents. Only 20 µL HPLC grade extraction solvent are used per extraction and the volume of internal standard is reduced through the integrated sprayer. Since the rinsing solution flows through separate channels, non LC-MS grade solvents can be used here. The extended newborn screening panel for MS detection therefore not only reduces the amount of solvents and labor, it also provides the foundation of good laboratory practice (GLP) by an integrated documentation system. The dried blood spots are directly eluted from the filter cards without any punching and are therefore traceable at all times during the analysis process. Each card is documented before and after analysis to ensure the highest standards of quality and documentation.

## Figures and Tables

**Table 1:** Intra-day and inter-day precision and R^2^ of calibrators.

Compound	Intra-Day *	Inter-Day **	R^2^	Compound	Intra-Day *	Inter-Day **	R^2^
	[%]	[%]			[%]	[%]	
Alanine	8.6	19.0	0.993	Valeryl-carnitine	3.9	12.8	0.999
Arginine	13.6	11.4	0.998	C5DC-carnitine	14.0	17.2	0.616
Aspartic acid	9.3	41.4	0.994	Hexanoyl-carnitine	4.3	11.6	0.999
Citrulline	15.6	18.5	0.999	Octanoyl-carnitine	14.0	18.7	0.996
Glutamic acid	8.2	8.5	0.999	Decanoyl-carnitine	11.8	17.4	0.971
Glycine	8.0	20.3	0.991	Lauroyl-carnitine	3.3	7.3	0.999
Leucine	3.4	11.4	0.999	Myristoyl-carnitine	8.4	19.3	0.875
Methionine	7.0	10.9	0.998	Palmitoyl-carnitine	3.6	5.8	0.997
Ornithine	10.0	10.6	0.999	Stearoyl-carnitine	2.7	5.6	0.999
Phenylalanine	5.5	13.8	0.997	Cortisol	15.2	18.2	0.999
Proline	4.3	11.7	0.995	21-Deoxy-cortisol	10.0	9.7	0.994
Tyrosine	4.5	6.3	0.997	11-Deoxy-cortisol	8.9	13.2	0.999
Valine	5.5	10.1	0.985	17-Hydroxy-progesterone	19.9	16.2	0.972
Carnitine	6.2	11.3	0.999	11-Deoxy-corticosterone	7.6	10.9	0.996
Acetyl-carnitine	5.7	6.8	0.998	Progesterone	11.5	13.5	0.992
Propionyl-carnitine	10.7	12.4	0.998	Androstene-dione	3.4	8.8	0.997
Butyryl- carnitine	5.7	9.0	0.983	Corticosterone	13.2	17.9	0.996

* Of first time point, level 2, ** Storage at 25 °C, level 2, R^2^ from day 1, level 1–3.

## Supplementary Materials

The following are available online at www.mdpi.com/2409-515X/4/1/2/s1.
