# Supplementary material for: Extended and Fully Automated Newborn Screening Method for Mass Spectrometry Detection
Source: Int J Neonatal Screen. 2017 Dec 29;4(1):2. doi: 10.3390/ijns4010002 (PMC7548895; doi:10.3390/ijns4010002)
Supplement: Supplementary file 1 [file IJNS-04-00002-s001.docx]

Article

Extended and Fully Automated Newborn Screening Method for Mass Spectrometry Detection

Stefan Gaugler^1^, Jana Rykl^2^, Irene Wegner^3^, Tamara von Däniken^4^, Ralph Fingerhut^4#^, and Götz Schlotterbeck^3,#,^*

^1^ CAMAG, Sonnenmattstr. 11, 4132 Muttenz, Switzerland; stefan.gaugler@camag.com

^2^ Shimadzu Schweiz GmbH, Römerstrasse 3, 4153 Reinach, Switzerland; jr@shimadzu.ch

^3^ University of Applied Sciences Northwestern Switzerland FHNW, Gründenstr. 40, 4132 Muttenz, Switzerland; irene.wegner@fhnw.ch

^4^ Swiss Newborn Screening Laboratory, Division of Metabolism and Children’s Research Center, University Children’s Hospital Zurich, Steinwiesstrasse 75, 8032 Zurich, Switzerland; Tamara.vonDaeniken@bluewin.ch (T.v.D.); Ralph.Fingerhut@kispi.uzh.ch (R.F.)

***** Correspondence: goetz.schlotterbeck@fhnw.ch; Tel.: +41-61-228-5476

**Table S1:** m/z transitions of all compounds.

| Name | Pre. | Quant. | Pre. 2 | Qual. | Qual.2 | Q1 | Q2 | Q3 |
| --- | --- | --- | --- | --- | --- | --- | --- | --- |
|  | [m/z] | [m/z] | [m/z] | [m/z] | [m/z] | [V] | [V] | [V] |
| Alanine | 90.2 | 44.2 |  |  |  | 10 | 12 | 19 |
| Alanine-^2^H_4_ | 94.2 | 48.2 |  |  |  | 10 | 12 | 19 |
| Arginine | 175.2 | 70.2 |  |  |  | 10 | 24 | 16 |
| Arginine-^2^H_7_ | 182.2 | 77.2 |  |  |  | 10 | 24 | 16 |
| Aspartic acid | 134.2 | 134.2 |  |  |  | 15 | 12 | 13 |
| Aspartic acid-^2^H_3_ | 137.2 | 75.0 |  |  |  | 16 | 16 | 27 |
| Citrulline | 176.1 | 113.1 |  |  |  | 10 | 16 | 25 |
| Citrulline-^2^H_2_ | 178.1 | 115.1 | 137.2 | 75.0 | 77.2 | 10 | 16 | 25 |
| Glutamic acid | 148.2 | 84.1 | 147.8 | 116.2 | 84.1 | 17 | 17 | 14 |
| Glutamic acid-^2^H_5_ | 153.1 | 88.2 |  |  |  | 17 | 18 | 19 |
| Glycine | 76.0 | 30.0 |  |  |  | 11 | 12 | 30 |
| Glycine-^13^C_3_, ^15^N_1_ | 79.0 | 32.0 | 79.0 | 31.1 | 15.2 | 10 | 16 | 28 |
| Leucine | 132.0 | 86.2 | 76.0 | 58.2 |  | 16 | 12 | 19 |
| Leucine-^2^H_3_ | 135.0 | 89.2 | 79.0 | 31.2 | 15.2 | 16 | 12 | 19 |
| Methionine | 150.1 | 104.1 |  |  |  | 18 | 14 | 22 |
| Methionine-^2^H_3_ | 153.1 | 107.0 | 153.1 | 89.1 | 121.3 | 18 | 13 | 18 |
| Ornithine | 133.2 | 133.2 |  |  |  | 16 | 12 | 27 |
| Ornithine-^2^H_6_ | 139.2 | 76.0 |  |  |  | 15 | 19 | 15 |
| Phenylalanine | 166.2 | 120.2 |  |  |  | 18 | 14 | 28 |
| Phenylalanine-^2^H_5_ | 171.2 | 125.2 | 139.2 | 122.2 | 43.1 | 18 | 14 | 28 |
| Proline | 116.2 | 116.1 |  |  |  | 14 | 13 | 23 |
| Proline-^2^H_7_ | 123.2 | 77.1 |  |  |  | 13 | 18 | 16 |
| Tyrosine | 182.1 | 123.1 |  |  |  | 10 | 18 | 24 |
| Tyrosine-^2^H_4_ | 186.1 | 127.1 | 123.2 | 81.1 | 49.2 | 10 | 18 | 24 |
| Valine | 118.2 | 72.1 |  |  |  | 14 | 13 | 17 |
| Valine-^2^H_8_ | 126.2 | 80.2 |  |  |  | 14 | 13 | 17 |
| Carnitine | 162.0 | 85.0 |  |  |  | 21 | 23 | 20 |
| Carnitine-^2^H_9_ | 171.0 | 85.0 |  |  |  | 10 | 19 | 17 |
| Acetylcarnitine | 204.0 | 85.0 |  |  |  | 20 | 25 | 20 |
| Acetylcarnitine-^2^H_3_ | 207.0 | 85.0 |  |  |  | 11 | 19 | 18 |
| Propionylcarnitine | 217.9 | 85.0 |  |  |  | 20 | 25 | 20 |
| Propionylcarnitine-^2^H_3_ | 221.0 | 85.0 |  |  |  | 12 | 23 | 18 |
| Butyrylcarnitine | 231.9 | 85.0 |  |  |  | 20 | 25 | 20 |
| Butyrylcarnitine-^2^H_3_ | 235.0 | 85.0 |  |  |  | 13 | 23 | 17 |
| Valerylcarnitine | 246.0 | 85.0 |  |  |  | 20 | 14 | 21 |
| Valerylcarnitine-^2^H_9_ | 255.0 | 85.0 |  |  |  | 14 | 24 | 17 |
| C5DC-carnitine | 276.2 | 85.0 |  |  |  | 15 | 26 | 19 |
| C5DC-carnitine-^2^H_6_ | 282.6 | 85.0 |  |  |  | 15 | 26 | 19 |
| Hexanoylcarnitine | 260.0 | 85.0 |  |  |  | 20 | 25 | 20 |
| Hexanoylcarnitine-^2^H_3_ | 263.0 | 85.0 |  |  |  | 13 | 22 | 14 |
| Octanoylcarnitine | 288.0 | 85.0 |  |  |  | 20 | 30 | 20 |
| Octanoylcarnitine-^2^H_3_ | 291.0 | 85.0 |  |  |  | 15 | 23 | 14 |
| Decanoylcarnitine | 316.0 | 85.0 |  |  |  | 10 | 24 | 17 |
| Decanoylcarnitine-^2^H_3_ | 319.0 | 85.0 |  |  |  | 10 | 24 | 17 |
| Lauroylcarnitine | 344.0 | 85.0 |  |  |  | 20 | 28 | 20 |
| Lauroylcarnitine-^2^H_3_ | 347.0 | 85.0 |  |  |  | 11 | 25 | 19 |
| Myristoylcarnitine | 372.2 | 85.0 |  |  |  | 19 | 30 | 20 |
| Myristoylcarnitine-^2^H_3_ | 375.0 | 85.0 |  |  |  | 11 | 27 | 17 |
| Palmitoylcarnitine | 399.9 | 85.0 |  |  |  | 12 | 28 | 18 |
| Palmitoylcarnitine-^2^H_3_ | 402.9 | 85.0 |  |  |  | 12 | 28 | 18 |
| Stearoylcarnitine | 427.9 | 85.0 |  |  |  | 13 | 29 | 17 |
| Stearoylcarnitine-^2^H_3_ | 430.9 | 85.0 |  |  |  | 13 | 29 | 17 |
| Cortisol | 362.8 | 121.0 | 362.8 | 327.2 | 309.2 | 20 | 27 | 21 |
| Cortisol-^2^H_4_ | 366.8 | 121.1 | 366.8 | 331.3 | 349.3 | 20 | 24 | 12 |
| 21-Deoxycortisol | 346.9 | 311.1 | 346.9 | 269.2 | 121.1 | 18 | 16 | 21 |
| 21-Deoxycortisol-^2^H_8_ | 354.9 | 319.3 | 354.9 | 125.2 | 180.2 | 11 | 17 | 22 |
| 11-Deoxycortisol | 346.9 | 109.0 | 346.9 | 97.0 | 79.2 | 20 | 30 | 20 |
| 11-Deoxycortisol-^2^H_5_ | 351.9 | 100.1 | 351.9 | 113.2 | 334.3 | 11 | 27 | 23 |
| 17-Hydroxyprogesterone | 331.4 | 97.0 | 331.4 | 109.1 |  | 17 | 25 | 16 |
| 17-Hydroxyprogesterone- ^2^H_8_ | 339.0 | 100.2 | 339.0 | 113.1 | 321.3 | 13 | 27 | 10 |
| 11-Deoxycorticosterone | 331.1 | 109.0 | 331.1 | 97.0 | 79.1 | 20 | 25 | 20 |
| 11-Deoxycorticosterone- ^13^C_3_ | 334.1 | 100.1 | 334.1 | 112.2 |  | 17 | 24 | 17 |
| Progesterone | 315.0 | 109.1 | 314.9 | 203.1 | 219.1 | 10 | 26 | 23 |
| Progesterone-^2^H_9_ | 324.0 | 100.0 | 324.0 | 113.0 | 306.4 | 20 | 25 | 20 |
| Androstenedione | 286.9 | 97.0 | 286.9 | 109.0 | 79.2 | 20 | 21 | 20 |
| Androstenedione-^13^C_3_ | 289.9 | 100.1 | 289.9 | 112.2 |  | 14 | 23 | 16 |
| Corticosterone | 347.2 | 329.1 | 347.2 | 121.2 | 311.2 | 18 | 16 | 22 |
| Corticosterone-^2^H_4_ | 351.1 | 333.2 | 351.1 | 121.2 |  | 18 | 17 | 21 |

The general settings of the mass spectrometer were as follows: nebulizing gas-2.8 L/min (N_2_), heating gas-9.5 L/min (N_2_), drying gas 9.5 L/min, positive mode, and source temperature 300°C. Labsolutions software (Shimadzu Kyoto, Japan) was used to operate the LC-MS/MS system. Pre. = precursor ion; Quant. = daughter ion for quantification; Pre.2 = precursor ion 2 (for qualifiers; Qual. = qualifier ion; Qual.2 = qualifier ion 2; Q1 = first quadrupole massfilter ; Q2 = quadrupole collision cell ; Q3 = second quadrupole massfilter

**T**able 2: Analyte concentration and levels.

|  | Spike | Solution | Solvent | Level1 | Level 2* | Level 3* | Level 4* |
| --- | --- | --- | --- | --- | --- | --- | --- |
|  | [μg/ml] |  |  |  | [μmol/l] | [μmol/l] | [μmol/l] |
| Alanine | 6200 | A | H_2_O | Endogenous concentration | 700 | 1400 | 2100 |
| Arginine | 1200 | A | H_2_O |  | 70 | 140 | 210 |
| Aspartic acid | 900 | A | H_2_O |  | 70 | 140 | 210 |
| Citrulline | 900 | A | H_2_O |  | 50 | 100 | 150 |
| Glutamic acid | 1000 | A | H_2_O |  | 70 | 140 | 210 |
| Glycine | 9000 | A | H_2_O |  | 1200 | 2400 | 3600 |
| Leucine | 5200 | A | H_2_O |  | 400 | 800 | 1200 |
| Methionine | 700 | A | H_2_O |  | 50 | 100 | 150 |
| Ornithine | 6600 | A | H_2_O |  | 500 | 1000 | 1500 |
| Phenylalanine | 2300 | A | H_2_O |  | 140 | 280 | 420 |
| Proline | 5800 | A | H_2_O |  | 500 | 1000 | 1500 |
| Tyrosine | 450 | A | H_2_O |  | 25 | 50 | 75 |
| Valine | 5900 | A | H_2_O |  | 500 | 1000 | 1500 |
| Carnitine | 1300 | A | H_2_O |  | 80 | 160 | 240 |
| Acetylcarnitine | 1600 | A | H_2_O |  | 80 | 160 | 240 |
| Propionylcarnitine | 170 | B | Methanol |  | 8 | 16 | 24 |
| Butyrylcarnitine | 30 | B | Methanol |  | 1.5 | 3 | 4.5 |
| Valerylcarnitine | 20 | B | Methanol |  | 0.7 | 1.4 | 2.1 |
| Glutarylcarnitine | 0 | B | Methanol |  | *n/a* | *n/a* | *n/a* |
| Hexanoylcarnitine | 10 | B | Methanol |  | 0.3 | 0.6 | 0.9 |
| Octanoylcarnitine | 20 | B | Methanol |  | 0.8 | 1.6 | 2.4 |
| Decanoylcarnitine | 10 | B | Methanol |  | 0.4 | 0.8 | 1.2 |
| Lauroylcarnitine | 10 | B | Methanol |  | 0.4 | 0.8 | 1.2 |
| Myristoylcarnitine | 30 | B | Methanol |  | 0.7 | 1.4 | 2.1 |
| Palmitoylcarnitine | 400 | B | Methanol |  | 10 | 20 | 30 |
| Stearoylcarnitine | 130 | B | Methanol |  | 3 | 6 | 9 |
| Cortisol | 20 | C | Methanol |  | 0.552 | 1.104 | 1.655 |
| 11-Deoxycortisol | 20 | C | Methanol |  | 0.577 | 1.155 | 1.732 |
| 21-Deoxycortisol | 20 | C | Methanol |  | 0.577 | 1.155 | 1.732 |
| Corticosterone | 20 | C | Methanol |  | 0.577 | 1.155 | 1.732 |
| Androstenedione | 20 | C | Methanol |  | 0.698 | 1.397 | 2.095 |
| 11-Deoxycorticosterone | 20 | C | Methanol |  | 0.605 | 1.210 | 1.816 |
| 17-Hydroxyprogesterone | 20 | C | Methanol |  | 0.605 | 1.210 | 1.816 |
| Progesterone | 20 | C | Methanol |  | 0.636 | 1.272 | 1.908 |

*in addition to endogenous concentration

**T**able S3:, Pipetting scheme for the preparation of the QC blood samples.

|  | Level 1 | Level 2 | Level 3 | Level 4 |
| --- | --- | --- | --- | --- |
|  | [μl] | [μl] | [μl] | [μl] |
| Spike A | 0 | 10 | 20 | 30 |
| Spike B | 0 | 10 | 20 | 30 |
| Spike C | 0 | 10 | 20 | 30 |
| H_2_O | 30 | 20 | 10 | 0 |
| Methanol | 60 | 40 | 20 | 0 |
| Blood | 910 | 910 | 910 | 910 |

**T**able S4: Camera report generated by the DBS-MS 500.

| Parameter | Value | Criteria |
| --- | --- | --- |
| Rack1, Card 4, Spot 1 | |  |
| Processing Results 2017-08-02 11:59:13 | |  |
| Barcode Number |  | Find CODE |
| Barcode Detected | TRUE | TRUE |
| Card Present | TRUE | TRUE |
| Card Orientation | TRUE | TRUE |
| Spot Detected | TRUE | TRUE |
| Spot Diameter | 6.81 | > 4 mm |
| Spot Area | 40.14 | > 15 mm^2^ |
| Spot Roundness | 95 | > 85 |
| Spot Shift X | -0.66 | n/a |
| Spot Shift Y | -0.77 | n/a |


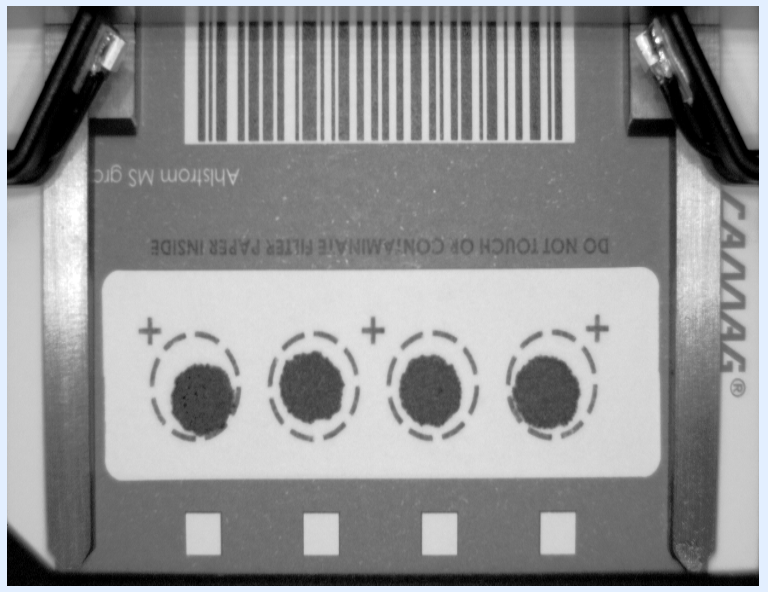


**Figure S1.** Quality control picture taken by the DBS-MS 500 integrated camera.

© 2017 by the authors. Submitted for possible open access publication under the
terms and conditions of the Creative Commons Attribution (CC-BY) license (http://creativecommons.org/licenses/by/4.0/).
